# Two Cases of Labor Complicated by Presence of Uterine Tumors, in One of Which Cæsarian Section Was Mooted

**Published:** 1869-09

**Authors:** C. C. F. Gay

**Affiliations:** Buffalo, N. Y.


BUFFALO

### Medical and Surgical Journal.

VOL. IX. SEPTEMBER, 1869. No. 2.

### Original Communications.

ART. I.—*Two cases of Labor complicated by presence of Uterine Tumors, in one of which Cæsarian section was mooted.* By C. C. F. Gay, M. D., Buffalo, N. Y.

Case 1st.—I was summoned in haste to visit, in the country, a woman, 45 years of age, American, the mother of several children born of natural labor, whose previous health had been good. A considerable interval having elapsed between this and the former pregnancy, it was, until quickening, believed that the suppression of menstruation was owing to the approach of the climacteric period. I found, on inquiry, that this woman had been abandoned, by an irregular practitioner, after an unfinished severe labor, extending over a period of thirty-six hours.

On examination, ascertained that it was a case of face presentation, the pains had nearly or quite ceased, and the woman scarcely had pulsation at the wrist.

I immediately administered Tr. Opii ʒss, and ordered brandy and beef tea, and dispatched a messenger for an assistant; but there being no time for delay, I applied the forceps, but all the traction I was able to make would not move the child; and, after unsuccessful trial, I performed the operation of craniotomy, diminishing the cranial diameter, again applied the forceps and delivered the child with ease. The patient never rallied but died four hours thereafter, apparently from exhaustion.

Digital examination, made before I attempted to deliver, induced me to think that the cervix uteri had been ruptured. The os was not in its entire circumference; symmetrically dilated, upon the left aspect, the dilated os ran off to a point or acute angle; the opening was V shaped, circular upon the right, and running to an acute angle, as if the os and cervix were slit down by a laceration. I, therefore, desired and obtained post-mortem examination, which revealed the fact that the cervix was not ruptured, but that the peculiar form or dilated shape of the os was dependent upon the presence of a fibrous tumor the size of my fist, located upon the body of the uterus, thus preventing the symmetrical contraction of all the fibers of the uterus, and normal dilation of the os.

*Remarks.*—There were no need of fatal result in this case had timely aid been rendered. Labor had been protracted far beyond the capacity or the physical strength of the patient to endure. Chloroform had not been used, but when I dispatched a messenger for assistance I also sent for chloroform, but neither arrived in time. The actual time consumed in my manipulations was one hour. I had delayed a few moments for opium and stimulation to take effect upon the patient, hoping that she might be made to rally sufficiently, by the aid of these remedies, to ensure safe delivery, but she was too nearly moribund when I first saw her for me to think of awaiting the arrival of an assistant, or the procurement of the anæsthetic.

Had the presentation been normal, the efforts of nature would undoubtedly have been sufficient for the expulsion of the child, notwithstanding the presence of the tumor. The head of the child had become firmly impacted, and no amount of strength that I was able to expend could move it, hence craniotomy was elected as the only remaining measure to be adopted, or else allow the woman to die undelivered. The peculiar and irregular shape of the dilated os may be regarded in the graved uterus as an unerring diagnostic sign of the presence of uterine tumor.

---

Case 2d.—Mrs. A., primipara, aged 32 years, American, a slim, delicately formed female, was found, on examination made after three months pregnancy, to have large uterine tumor. She was attended by Dr. Hutchins, who has kindly permitted me to make report of this interesting and instructive case.

During the first three or four months of this her first pregnancy, she was seen and examined, at different times, by Drs. Lothrop and White, both of whom wisely advised the induction of premature labor, but the patient would not give her assent, though told that in the event of her going on to the completion of her full period of utero-gestation, the operation of Cæsarian section would become a necessity, this being the only mode of delivery that would seem to give promise of success, the peril of which would be much greater than that incident to the induction of premature labor.

This woman was taken in labor at full term, at 7 o'clock on the evening of July 23d, 1869, Dr. Hutchins in attendance. Labor pains increased in frequency and strength, and the case progressed in a manner not unlike any normal labor for several hours. On digital examination, Dr. H. reports that the os did not dilate in a circle but in a straight line running laterally, and that a foot presented. He siezed the foot, and after several pains it was brought down and occupied position outside the vulva.

Drs. Pratt, Brown and myself were now, at 1½ A. M., sent for by Dr. H. to assist him in the delivery, and to determine in the outset the feasibility of the operation of Cæsarian section, and if found advisable to perform it at once. After the patient was anæsthetized I made examination by introducing my entire hand within the vagina, for a short distance, when it was arrested by the tumor, which felt hard and unyielding, and occupied the pelvic cavity, posteriorly; beyond this tumor there appeared only space for the child's thigh, which completely occupied and filled up the space, the foot resting beyond the vulva, as above stated; the other foot could be felt with the index and middle fingers, and during pains could be grasped but not brought down. By permission of Dr. Hutchins, I made traction at the recurrence of several labor pains, so strong that I felt, if little more strength was expended, that I should dislocate the child's leg, and yet without any perceptible progress made towards expulsion of the body of the child. It seemed advisable that every possible effort should be made to deliver the woman without resort to gastrotomy, hence, when I felt some fatigue from my efforts, I was relieved by Dr. Pratt, who advised the use of and gave ergot, and suspended the use of chloroform. He succeeded, after an hour's effort, alternating with Dr. Hutchins, in delivering the body of the child, but the head remained firmly impacted despite all the efforts of these two gentlemen, after prolonged trial.

Again giving chloroform, I made trial and succeeded in reaching the mouth of the child, and with my two fingers thrust within the mouth found all efforts unavailing. The other gentlemen present all made unsuccessful trial in this way; by request of Dr. H., I then attempted to perform craniotomy, the patient again under influence of chloroform, but there was no point upon the cranium nor through the mouth that was penetrable to the perforator. I should, however, be more correct by stating that there was no point of the child's cranium that could be reached with the perforator other than in a line parallel to the cranium, therefore trial was made to perforate through the roof of the mouth, and on failure to reduce the cranial capacity, as was hoped, I immediately applied the long forceps, by the aid of which I was enabled, with the use of all the strength I could expend, aided by Dr. Brown, who also siezed the forceps with one hand, and Dr. Pratt making traction upon the child, in delivering the woman at 5 o'clock, A. M., of a still-born, well-formed male child.

August 9th.—The woman has been doing well and has sat up once. She has laceration of the recto-vaginal septum, which she is to have relieved by operation.

*Remarks.*—This case is suggestive of two questions, the correct answering of which is of considerable medical importance.

The first question has reference to the relative value of the Cæsarian Section, and the mode of delivery adopted.

The second question has reference to the relative value of the life of the mother and child.

In regard to the former of these two questions, the result of the case would seem to have furnished satisfactory answer. We are certainly not authorized to perform Cæsarian Section when delivery of a dead child may be accomplished through the natural outlet of the maternal organs.

Dr. Robert Barnes says: "The most frequent condition that renders the Cæsarian Section necessary is *deformity with contraction of*

*the pelvis.*" "The next most frequent condition or cause is the presence of tumors of various kinds growing into the pelvis, such as bony or malignant tumors springing from the wall of the pelvis, tumors of the ovary descending into the pelvic cavity and getting fixed there."

A case is recorded in the *Medical Times and Gazette* for 1844, in which the operation became necessary from the pelvis being filled up with an enormous *hydatid cyst* springing from the liver.

Other exceptional cases, chiefly remarkable on account of their extreme variety, have been observed. Atresia of the cervix uteri and vagina may be so extensive and unyielding that the Cæsarian Section may be less hazardous than the attempt to open up a canal through the cicatricial tissues. "We are sometimes driven to the operation, says Dr. Barnes, after having exhausted other modes of proceeding—for example, when craniotomy may have failed to deliver."

Within the catalogue of the risks attending the operation, Dr. Robert Barnes enumerates the following: secondary shock and peritonitis, hemorrhage, and septicæmic puerperal fever.

Although Dr. Robert Barnes makes mention of "tumors of various kinds growing into the pelvis," he does not make mention directly, though this may be inferred or implied in what he actually does state, of large fibrous or malignant growths located upon the body of the uterus, filling up the pelvic cavity, and leading to the necessity of the Cæsarian Section.

I conclude from the quotations made above that Dr. Barnes would consider the case of the woman who is the subject of this discussion, as one in whom the conditions were present such as would call for the Cæsarian Section.

As this woman was delivered there would seem to be no need of offering an opinion; but should I presume to venture upon the expression of an opinion, I should say this much, that with this woman undelivered, with the attendant difficulties to overcome, and uncertainty of overcoming these difficulties after protracted efforts, I should not hesitate to decide in favor of the Cæsarian Section. After the knowledge I now possess, *i. e.,* with the full knowledge that the woman is delivered with safety to herself, I must, in this case, not hesitate to say that there was certainly not much preference in choice between the two methods of delivery. The danger to the woman under either method of proceeding may be regarded as very nearly equal, while the danger to the child in the two methods was very unequal. Cæsarian Section might probably have saved both mother and child, the other method of delivery was sure to destroy the child.

The next question to be discussed briefly is that of the relative value of mother and child. Were it possible to concede greater value to the life of the offspring than to that of the mother, then the first question, viz., the relative value of the two methods of delivery would be answered. If the duties to be performed by the accoucheur were directed to the saving the life of the child, as duties paramount to the safety of the mother, gastrotomy would take precedence without question or equivocation; but I am not willing to assert that there can arise any contingency to the family or state which would justify the sacrifice of the mother's life in order to save that of the child. The edict has gone forth into all the dark and benighted, as well as illuminated, places of the earth, to save the mother even though you destroy the child. I am not, therefore, to make any attempt to gainsay the propriety of this edict, which has come down to us with the sanction of the highest authority, and which has long been accepted by the profession as the infallible rule.

I do not desire to call this infallible rule in question, or to seek exceptions to it, although, if it would not be an unwarrantable innovation, I would barely suggest that since there are exceptions to all general rules in almost every thing in the universal realm of nature, that, therefore, in the case under discussion, there is furnished, in estimating the valuation of human life, an exception to the long established rule.

The case before us is one wherein, after severest struggles, attended by many doubts and uncertainties as to successful result, on the part of the medical attendants, the patient was delivered of a dead child.

The mother survives with a recto-vaginal laceration, and with two tumors—one anteriorly, which can be felt distinctly through the abdominal walls, on palpation—and another, much larger in size, occupying the uterus posteriorly, both of which appear to be fibrous in their texture.

The laceration can be remedied by an operation, but the tumors cannot be removed, but must remain, if not to destroy the woman's life or health, to serve as a sad warning against ever again becoming subjected to conception and the perils of child bearing. Under physical disabilities such as these, human life becomes shorn of much of its value, prospects of longevity cannot be accounted as good, neither can any degree of comfortable health be assured.

Compare this woman's prospects of longevity, her usefulness and ability to comply with the Divine injunction, with those of a healthful, well-formed male child, arrived at maturity, and the relative valuation of the life of the former may be made to dwindle into insignificance, and almost nothingness, in comparison with that of the latter. But considerations such as these, whether weighty or not, will probably never be seriously thought of or acted upon, and for humane reasons never should be seriously thought of or acted upon by the physician in his repeated trials and struggles for the preservation of human life, and are only called up and discussed here in this connection on account of their supposed medico-legal bearing in some prospective case that may arise.

History, I believe, records but one instance of a human monster who desired death of the mother so that the life of the child could be preserved.

On the 12th of October, 1537, Jane Seymour, wife of King Henry the Eighth, was delivered of a son, after a labor so dangerous that the physicians, apprehensive that to save both lives would be impossible, left it to the option of the King, who ordered the wife and mother to be sacrificed, if need be, in order to save the child.

This same question was also put, as is the custom in royal accouchments, by the medical attendants of the Princess Charlotte; and the question was, in this instance, answered on the christian view of the subject, but in vain. A protracted labor resulted in the death of the Princess and her child. The result weighed so heavily upon the mind and spirits of Sir Richard Croft, the attendant in chief, that he committed suicide.

Napoleon possessed the great virtue—although desiring offspring above all things else, sacrificing Josephine with hope of having his desire gratified—of deciding this important question upon humane principles.

That Royal attendants should, by the seductions of place and patronage, became so mercenary as ever to consent to submit to any one, however exalted in station, the question of which life to preserve, is, in my view, so utterly revolting, that I should expect any medical attendant, who was possessed of ordinary sensibility, whether successful or not in saving one or both lives, to go immediately and do as Sir Richard Croft did—commit suicide.

These remarks upon this interesting case of midwifery would be incomplete did I not, in this place, make allusion to the presentation.

Had the vertex presented, craniotomy might, or might not, have been easily performed. It might have been impracticable on account of the want of room, which was occupied by the tumor, which served to reduce the conjugate diameter to its minimum. Another cause which would have made craniotomy, in vertex presentation, impracticable, would have been found on trial, in the fact of the abnormal dilatability of the os, and the absence of any progress whatever of the child's head through it. It may, therefore, have been a fortunate circumstance that the breach presented.

I must not close these comments without giving credit to whom credit is due. To the persevering efforts of Drs. Pratt and Hutchins this woman is indebted for her safe delivery. The latter gentleman, however, thought Cæsarian section the only practicable method of delivery, and in this opinion I fully concurred. I arrived at this conclusion in this wise. Having made traction upon the child's leg, during several labor pains, almost sufficiently strong to tear the limb from the body without perceiving any advance made by my efforts, I at once concluded, that should the body in this way be delivered, a consumation of which I had no doubt, that the head would remain undelivered. These fears of mine came very near being realized. I have, heretofore, performed craniotomy with the greatest facility, but am confident, after trial, that this was a case that would not allow of the time necessary to be consumed to perform it, admitting that it could have been made at all.

On yesterday, September 2d, I assisted Dr. Hutchins in the operation for incomplete laceration of the perineum of Mrs. A., and have no doubt the operation will prove successful.

